# When (distant) relatives stay too long: implications for cancer medicine

**DOI:** 10.1186/s13059-016-0906-3

**Published:** 2016-02-24

**Authors:** Diego Chowell, Amy M. Boddy, Diego Mallo, Marc Tollis, Carlo C. Maley

**Affiliations:** Biodesign Institute, Arizona State University, Tempe, Arizona 85281 USA; Simon A. Levin Mathematical, Computational and Modeling Sciences Center, Arizona State University, Tempe, Arizona 85281 USA; Department of Psychology, Arizona State University, Tempe, Arizona 85281 USA; Center for Evolution and Cancer, University of California San Francisco, San Francisco, California 94158 USA

## Abstract

Whole-genome analyses of human medulloblastomas show that the dominant clone at relapse is present as a rare subclone at primary diagnosis.

The extent of intratumor heterogeneity and how treatment affects clonal evolution of cancer is an emerging theme in the field of cancer biology. In the era of targeted therapy, the presence of pre-existing treatment-resistant subclones within the tumor is a critical barrier to achieving durable responses. Targeted drugs result in a significant clinical improvement if they are directed at the founding clonal mutations shared by all of the billions of cells in a tumor. However, growing evidence indicates that tumors also contain many subclonal mutations which are often present in only a few cancer cells [1–3]. These subclones are derived from the founding clone and are established by the additional mutations they acquire, which are not present in the bulk of the tumor. Importantly, many subclonal mutations cannot be detected because their abundance falls below the detection limit of standard genome or exome sequencing techniques [1]. In the case of solid tumors, spatial heterogeneity and tumor sampling errors can also lead to subclonalmutations being missed [1]. However, subclonal mutations can be clinically relevant [2, 4]. For example, in patients with chronic lymphocytic leukemia, the presence of subclonal populations impacts prognosis and clinical outcome [2]. Recent studies have also revealed that tumor cells corresponding to the relapse clone are often present as minor subclones within the primary tumor before the initiation of therapy, which suggests that genetic abnormalities contributing to recurrence are selected for during treatment [5–8].

In a new study, Morrissy and colleagues [9] study clonal evolution in relapsed medulloblastoma. By analyzing whole-genome sequencing (WGS) of 33 pairs of human diagnostic and post-therapy medulloblastoma samples, the authors show that the dominant clone in the recurrent tumor is often highly divergent from the dominant clone in the primary tumor. These results were also corroborated in a murine medulloblastoma model. The authors concluded that the dominant clone at recurrence arose through clonal selection of a pre-existing minor subclone present at diagnosis (Fig. 1). This study represents an important advance in understanding the clonal dynamics of recurrence in medulloblastoma.

**Fig. 1:**
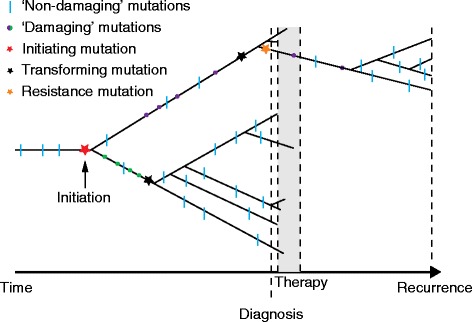
A timeline of neoplastic progression, therapy, and recurrence. We illustrate a hypothetical example of clonal expansion of neoplastic cells over time (*x*-axis). A cell lineage accumulates somatic mutations (and other genetic and epigenetic alterations), one of which initiates carcinogenesis (*red star*). These somatic mutations are shared across all neoplastic cells. During neoplastic progression, two separate lineages accumulate driver damaging mutations (*green and purple circles*) that lead to transformation events. A damaging mutation is defined as a nonsynonymous mutation that is predicted to have a functional effect. We define a transformation (*black star*) as the last mutational hit to make the clone malignant. During therapy most of the lineages are eliminated. However, a rare subclone at diagnosis with a resistance mutation (*orange star*) survives therapy and may continue to expand until reaching a detectable population size (recurrence). This hypothetical scenario reflects the 41.6 % of cases in which Morrissy and colleagues [9] did not find common damaging mutations between diagnosis and recurrence samples

## Recurrent tumors are very different from tumors at diagnosis and should be biopsied

An important consequence of the clonal nature of cancers is the spatial and temporal variation in the clonal composition within both primary and relapsed tumors, which makes the identification of trunk mutations of a specific tumor type a challenging problem. Morrissy and colleagues found that in 75 % of cases, the mutations that appeared to be clonal (“trunk mutations”) at diagnosis were either absent at relapse or not present in all the neoplastic cells. Previous studies have analyzed clonal evolution of recurrence in different cancers [2, 5, 6]. For example, in pediatric patients with acute lymphoblastic leukemia (ALL) it has been observed that there is a significant increase in the average number of copy number alterations per case in relapsed B-cell ALL compared with the matched diagnostic sample [5]. Morrissy and colleagues [9] performed WGS on matched diagnosis and relapse samples from 33 pediatric cases. In 13 out of 15 patients with matched germline DNA, there was on average a fivefold increase in the somatic mutational burden at recurrence. Furthermore, they found that, on average, only less than 12 % of somatic single nucleotide variants (SNVs) and indels were present in both the diagnostic and the relapse samples, demonstrating a substantial genetic divergence at relapse. It is worth mentioning that relapse samples also had an increased proportion of transversions, presumably due to DNA damage induced by the radiation therapy. They also corroborated these findings in a mouse medulloblastoma model, concluding that, similar to human medulloblastoma, there is a very small overlap (<5 %) of genetic events that appear to be in all of the cancer cells between the matched diagnostic and recurrent tumor samples. This implies that systematic multiregion sampling before treatment and at the time of recurrence is essential both to better understand clonal evolution and for the development of effective targeted therapies.

## Subclonal diversity makes tumors more evolvable and robust to interventions

Recent studies have identified rare subclones within the tumor that harbor resistance mutations before the initiation of therapy [5–8], which suggests that standing subclonal variation within a tumor may be the most important factor for the evolution of acquired resistance to treatment. In natural systems, it is well-studied that populations can adapt to novel environments in two different ways: selection acting on pre-existing genetic variants and selection acting on new mutations. Adaptation from standing genetic variation is likely to be faster than from novel mutations, not just because beneficial mutations are immediately available in the new environment, but also because they might start at higher frequencies. Morrissy and colleagues [9] found that the majority (60.5 %) of damaging (i.e., non-synonymous variants predicted to have a functional impact on the protein) clonal mutations present in the primary tumor sample consistently decreased in prevalence after therapy, and either became subclonal (25.9 %) or disappeared completely (34.6 %). Additionally, only 25 % of patients retained the full set of clonal SNVs after therapy, with most cases having no retention (41.6 %) or partial retention (33.3 %) of damaging clonal mutations. Importantly, all relapses shared somatic mutations with the primary tumor and therefore shared a common origin. This indicates that in 41.6 % of cases, the tumor was initiated by nondamaging mutations, which highlights the importance of considering other genetic modifications (e.g., mutations on non-coding regions, epigenetics, or copy number aberrations) both to study tumor evolutionary dynamics and to identify new candidate drivers. Taken together, these findings indicate that the majority of relapsed tumors had a clear relationship to the medulloblastoma clone at diagnosis, either arising through clonal evolution of the diagnostic dominant population or arising from an ancestral subclone, which was not the predominating clone at diagnosis.

## Distant relatives may never leave

A common model of clonal evolution in neoplastic progression envisions driver mutations causing clonal expansions that sweep through the neoplastic cell population and reach fixation (100 % frequency), driving other clones extinct. If such a mutation did reach fixation, it would appear as a trunk mutation, present in all the neoplastic cells. Morrissy and colleagues [9] provide evidence that such clonal expansions rarely reach fixation, and that distant relative clones remain in the neoplasm at very low frequency, below the threshold for detection at diagnosis. The authors performed ultradeep sequencing of patient-specific SNVs from 20 cases. Sixteen out of the 20 patients had evidence of rare subclones initially present at frequencies less than 5 % before the initiation of treatment. The lack of evidence of rare subclones in the remaining four patients may be simply due to sampling and spatial heterogeneity, or limits of detection of ultradeep sequencing itself [1]. This finding is consistent with previous studies [5–8]. For example, it has been shown that colorectal tumors that are wild type for *KRAS* are often sensitive to targeted epidermal growth factor receptor (EGFR) blockade; however, *KRAS* mutations become detectable at the time of acquired resistance to this targeted drug. An analysis of the evolutionary dynamics of cancer cells with *KRAS* mutations in patients with colorectal cancer after treatment demonstrated that these mutations had been present before the initiation of EGFR blockade, in rare subclones harboring approximately thousands of cancer cells [6]. Additional evidence for these rare subclones has been provided by the detection of low levels of *KRAS* mutations by sensitive PCR technology in patients with colorectal cancer who were found to be wild type for *KRAS* by standard methods [1]. Therefore, with the advent of more powerful sequencing methods in the future, determining the presence of treatment-resistant subclones within a tumor at the time of diagnosis will be crucial to decide how single therapeutic agents could be combined in order to control or suppress clonal evolution.

## New approaches to managing cancers

How can we deal with so much standing variation in a tumor at diagnosis, and the resulting evolvability of the tumor? In many cases, we may assume that a resistant clone is already present at diagnosis. The standard response has been to combine drugs in the hope that there are no cancer cells present that are resistant to all the drugs in the cocktail. While multidrug cocktails typically extend survival longer than monotherapy, resistance still evolves. Evolutionary theory suggests that we may slow or even avoid the evolution of resistance by using cytostatic drugs over cytotoxic drugs, by targeting the evolvability of the tumor itself by lowering the mutation rate and extending the generation time of the cancer cells, attempting to maintain control of the tumor by keeping some sensitive cells alive (“adaptive therapy”), or by selecting against the resistant clones [10]. At the very least, we must biopsy tumors at recurrence because they are likely to be quite different from the tumor that was biopsied at diagnosis.

## Abbreviations

ALL: acute lymphoblastic leukemia
EGFR: epidermal growth factor receptor
SNV: single nucleotide variants
WGS: whole-genome sequencing
